# FE65 Recognition Rewires the Dynamic Contact Network of the Amyloid Precursor Protein Intracellular Domain

**DOI:** 10.1021/acs.jpcb.6c04695

**Published:** 2026-10-23

**Authors:** Nabanita Mandal, Marie Skepö

**Affiliations:** † Division of Computational Chemistry, Department of Chemistry, Science for Life Laboratory, 5193 Lund University, P.O. Box 124, 221 00 Lund, Sweden; ‡ NanoLund, 90825 Lund University, Box 118, 221 00 Lund, Sweden

## Abstract

The amyloid precursor protein intracellular domain (AICD) is a short, intrinsically disordered signaling fragment that mediates interactions with adaptor proteins involved in APP trafficking and transcription-associated regulation. Among these partners, FE65 recognizes the conserved YENPTY-containing region of AICD, yet the ensemble-level mechanism underlying this interaction remains unclear. Here, we use molecular dynamics simulations to compare unbound and bound AICD together with YENPTY-motif mutant variants. In the unbound state, AICD samples a heterogeneous conformational ensemble dominated by disordered states while retaining transient local structure and recurrent intramolecular contacts. Upon FE65 binding, AICD does not collapse into a single rigid conformation; instead, its conformational ensemble is redistributed toward interaction-compatible substates. Residue-resolved contact analyses identify localized interaction hotspots within and adjacent to the YENPTY region, including aromatic and polar residues that dominate the bound-state contact network. Mutations within the YENPTY motif alter the balance between compact and extended conformations while preserving the intrinsically disordered character of the peptide. Together, these results support a recognition mechanism in which FE65 binds a partially organized AICD ensemble via localized interaction hotspots and ensemble reweighting, rather than through complete disorder-to-order folding. This work provides a molecular framework for understanding APP intracellular-tail recognition and its regulation by sequence-localized perturbations. More broadly, these findings illustrate how intrinsically disordered motifs can achieve specific molecular recognition through redistribution of dynamic contact networks without undergoing complete disorder-to-order transitions.

## Graphical abstract

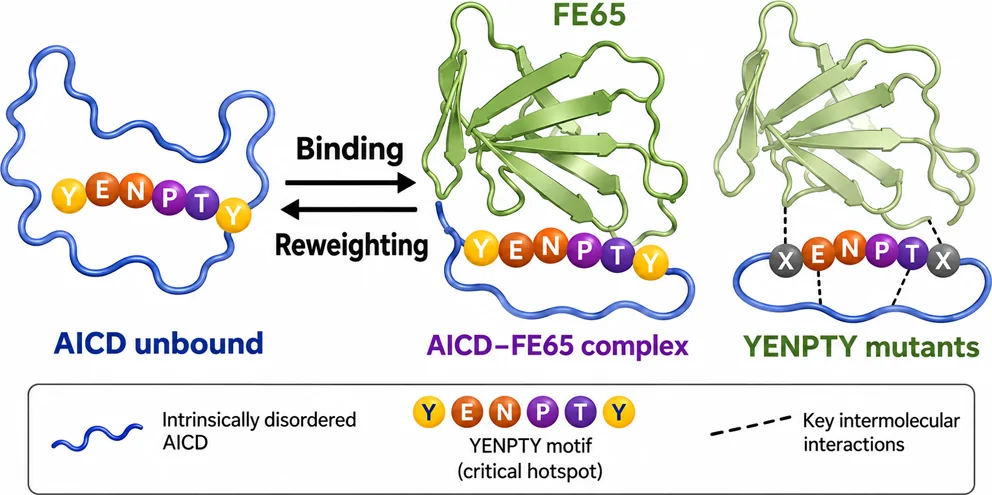

## Introduction

Amyloid precursor protein (APP) is a type I transmembrane glycoprotein that has been studied extensively because of its central role in Alzheimer’s disease through proteolytic pathways that generate amyloid-β peptides.^1^ Beyond amyloid-β production, APP processing also releases the amyloid precursor protein intracellular domain (AICD), a short cytosolic fragment implicated in intracellular signaling, transcription-associated regulation, and adaptor-protein recruitment.^2,3^ These functions suggest that APP biology extends beyond peptide generation and includes molecular recognition events mediated by the intrinsically flexible intracellular tail.

AICD is challenging to characterize structurally because of its short length, conformational heterogeneity, and intrinsically disordered nature.^4,5^ Rather than adopting a unique stable fold in solution, AICD is expected to populate an ensemble of rapidly interconverting conformations with transient local structure.^6^ Such behavior is characteristic of intrinsically disordered proteins and peptides, whose biological activities are often governed by ensemble properties, sequence-localized interaction motifs, and conformational redistribution upon binding.^7^ For AICD, this conformational plasticity is likely central to its ability to interact with multiple adaptor proteins and participate in context-dependent signaling processes.^8^

One of the best-characterized binding partners of AICD is FE65, an adaptor protein containing two conserved tryptophan (W) residues (WW domain) and two phosphotyrosine-binding domains, PTB1 and PTB2.^9^ FE65 recognizes APP/AICD primarily through its PTB2 domain and the conserved YENPTY-containing region of the APP intracellular tail.^10^ This interaction has been associated with AICD stabilization, nuclear localization, and downstream transcription-related signaling. Structural studies have further shown that the FE65–AICD interface is unusually extended for a PTB-mediated interaction and involves contacts outside the canonical YENPTY motif.^11,12^ Thus, the FE65–AICD complex provides an important model for understanding how a short disordered peptide is recognized through both motif-specific and extended intermolecular contacts.

Experimental studies have established that AICD interacts with FE65 and have defined several biological consequences of this association, but they do not fully resolve how the unbound AICD ensemble is reorganized upon binding, which residues dominate recognition, or how much flexibility is retained in the bound state.^13^ These questions are particularly relevant for intrinsically disordered systems, where binding often involves a combination of conformational selection, induced structural rearrangement, and residual disorder rather than complete folding into a rigid complex.^14,15^ In this framework, recognition is controlled by dynamic contact networks and residue-level interaction hotspots rather than by uniform stabilization across the entire sequence.

The conserved YENPTY motif is of particular interest because it plays a central role in APP cytoplasmic-tail recognition, internalization, and adaptor recruitment. Reported mutations and motif-disrupting variants, including N684A, ΔNPTY, and multiresidue YENPTY substitutions, have been used experimentally to perturb APP adaptor binding and YENPTY-dependent cellular functions.^16^ Incorporating these perturbations into molecular simulations provides a route to connect experimentally observed functional effects with residue-resolved changes in conformational organization and binding interactions. In this way, mutation-based systems can reveal whether recognition is dominated by the canonical motif alone or by a broader network of contacts distributed across the AICD sequence.^17^

In the present work, we use molecular dynamics (MD) simulations to characterize unbound and bound AICD and to examine how YENPTY-region perturbations alter its conformational and interaction landscapes.^18^ The systems considered include unbound AICD, bound AICD, and the experimentally motivated N684A, ΔNPTY, and multiresidue YENPTY mutant. By comparing these systems, we determine how FE65 binding reshapes the AICD ensemble, identify residue-level hotspots that contribute to intramolecular organization and intermolecular recognition, and assess how motif disruption modifies the balance between flexibility and binding-competent structure.^19^ This work provides an ensemble-based molecular description of AICD recognition and clarifies how localized sequence perturbations within the YENPTY region can influence APP intracellular-tail signaling.

## Methodology

### System Preparation

All MD simulations were performed in GROMACS 2024.3 ^20^ to characterize the conformational ensembles of unbound and bound AICD, and in YENPTY-motif mutant. The AICD sequence corresponded to the APP intracellular region containing the conserved YENPTY recognition motif. The FE65-bound system was prepared using the experimentally determined crystal structure of human APP AICD in complex with the PTB2 domain of FE65 (PDB ID: 3DXC), resolved by X-ray diffraction at 2.10 Å resolution.^12^

Prior to simulation, one FE65-AICD complex was extracted from the experimental structure. Crystallographic solvent molecules and nonprotein heteroatoms were removed, and hydrogen atoms were added according to standard protonation states at physiological pH. The FE65-bound system was then prepared using the AICD peptide in complex with the FE65 PTB2 domain. Three YENPTY-motif variants were constructed from the wild-type AICD sequence. The sequences used for the wild-type and mutant AICD systems are presented in Figure 9. For all mutant systems, substitutions or deletions were introduced into the wild-type AICD coordinates while retaining the surrounding AICD sequence context. The modified structures were visually inspected, and the local geometry around the mutation or deletion site was relaxed by energy minimization prior to production simulations.

Each system was placed in a periodic dodecahedral simulation box with a minimum solute–box distance of 1 nm. The systems were solvated using the TIP4P-D water model,^21^ neutralized with the appropriate number of counterions, and supplemented with additional Na^+^ and Cl^–^ ions to obtain an ionic strength of 10 mM NaCl. The 10 mM NaCl condition was selected to examine the intrinsic conformational preferences of AICD under weak electrostatic screening and to maintain consistency across the AICD within the FE65 trajectory, in the unbound state, FE65-bound AICD, and mutant systems. Because this ionic strength is lower than physiological salt concentrations, the resulting ensembles should be interpreted as low-ionic-strength conformational ensembles rather than direct representations of the cytosolic ionic environment.

All systems were described using the AMBER99SB-ILDN^22^ force field in combination with the TIP4P-D water model. AMBER99SB-ILDN was selected because it includes improved side-chain torsion potentials relative to earlier AMBER99SB variants and has been widely used for peptide and protein simulations. TIP4P-D was used because it improves the description of protein–water interactions and has been shown to reduce the excessive compaction often observed for intrinsically disordered proteins with some standard water models. Thus, the AMBER99SB-ILDN/TIP4P-D combination provides a suitable framework for comparative simulations of the short and conformationally flexible AICD peptide. Nevertheless, because AICD is intrinsically disordered, the simulated ensembles remain force-field dependent and were interpreted comparatively across the different systems.

### Molecular Dynamics Simulations

Energy minimization was first performed using the steepest-descent algorithm. The minimized systems were subsequently equilibrated in three stages: 0.5 ns in the *NVT* ensemble, followed by 0.5 ns in the NPT ensemble and an additional 1.0 ns in the NPT ensemble. Here, *NVTP* denotes a constant number of particles, volume, temperature, and pressure. Positional restraints were applied to the peptide during all equilibration steps.

Production MD simulations were then performed under periodic boundary conditions. For each system, five independent production replicas of 1 μs were generated, yielding a total nominal sampling time of 5 μs per system. Coordinates were saved every 1 ps. The first 300 ns of each replica were excluded as equilibration, and the remaining 700 ns were used for analysis. Thus, the total analyzed sampling time was 3.5 μs per system. Independent replicas were initialized separately to improve conformational sampling and to assess the robustness of the ensemble-based conclusions.

The temperature was maintained at 298 K using the Nosé–Hoover thermostat^23^ with a coupling time of 1.0 ps. The pressure was controlled isotropically using the Parrinello–Rahman barostat^24^ with a coupling time of 5.0 ps and a compressibility of 4.5 × 10^–5^ bar^–1^. Long-range electrostatic interactions were treated using the particle-mesh Ewald method^25^ with cubic interpolation and a grid spacing of 1.6 Å. Short-range nonbonded interactions were treated using the Verlet cutoff scheme and truncated at 12 Å. Bonds involving hydrogen atoms were constrained using the LINCS algorithm,^26^ allowing an integration time step of 2.0 fs.

Before analysis, all trajectories were corrected for periodic boundary conditions, centered, and aligned to remove global translation and rotation. For unbound AICD and the mutant systems, trajectory alignment was performed using the peptide backbone or Cα atoms. For the FE65–AICD complex, trajectories were aligned using the backbone atoms of the complex, and AICD-specific properties were extracted from the aligned trajectories.

### Root-Mean-Square Fluctuation Analysis

For Root-Mean-Square Fluctuation Analysis (RMSF)^20^ uncertainty estimation, the first 300 ns of each 1 μs production trajectory were excluded from the analysis. The remaining 700 ns of each equilibrated replica were divided into five nonoverlapping blocks of 140 ns. RMSF was calculated independently for each block after structural alignment. The reported residue-wise RMSF corresponds to the mean across all 25 replica–block estimates, and the uncertainty is reported as the standard error of the mean

$${\mathrm{SEM}}_{i}=\frac{s_{i}}{\sqrt{N}}$$

where *s*_*i*_ is the standard deviation of the RMSF values for residue *i* across the replica–block estimates and *N* = 25 is the total number of replica–block estimates.

### Radius of Gyration

The global compactness of the simulated systems was evaluated from the radius of gyration, *R*_*g*_,^20^ defined as

1 $$R_{g}={\left[\frac{\sum_{i}m_{i}{\left|r^{*}i-r^{*}\mathrm{COM}\right|}^{2}}{\sum_{i}m_{i}}\right]}^{1/2}$$

where *m*_*i*_ and **r** * *i* are the mass and position of atom *i*, respectively, and **r** * COM is the center of mass of the selected atoms. For unbound AICD and the mutant systems, *R*_*g*_ was calculated using the peptide atoms. For the FE65–AICD complex, *R*_*g*_ was calculated for the complete bound assembly. Probability distributions of *R*_*g*_ were generated from the production trajectories to compare compact and extended conformational populations.

### Secondary-Structure Analysis

Secondary-structure propensities were calculated for each residue over the production trajectories using DSSP.^27^ The analyzed secondary-structure classes included α-helix, β-bend, β-bridge, β-strand, 3_10_-helix, π-helix, polyproline II-like helix, turn, and unordered conformations. Residue-resolved secondary-structure probabilities were obtained by averaging the assigned secondary structure of each residue over all analyzed trajectory frames.

For the YENPTY-motif mutants, the relationship between chain compactness and secondary-structure formation was examined by calculating secondary-structure fractions as a function of *R*_*g*_. The trajectory frames were grouped into *R*_*g*_ bins, and the fractional populations of helix, β-sheet, and coil conformations were calculated within each bin. This analysis was used to determine whether changes in compactness were associated with the formation of ordered secondary structure or with the expansion of a predominantly disordered ensemble.

### Residue Contact Analysis

To isolate the effect of FE65 binding, the FE65–AICD trajectory was partitioned into bound and unbound states based on the persistence of contacts at the FE65–AICD interface. A residue pair was considered to be in contact when the minimum distance between any pair of non-hydrogen atoms was less than or equal to 8 Å. Persistent interfacial contacts were defined as the presence of at least *N*_c_ FE65–AICD residue contacts that remained continuously present for a minimum duration of τ_p_. Frames satisfying this criterion were assigned to the bound state, whereas frames that did not satisfy it were assigned to the unbound state. Brief interruptions shorter than τ_gap_ were treated as part of the same contact event to avoid classifying transient distance fluctuations as binding or unbinding transitions.

All subsequent analyses of the FE65–AICD system were performed separately for the two trajectory subsets, allowing direct comparison of the AICD conformational ensemble in the presence and absence of persistent FE65 interactions within the same simulation. Unless otherwise stated, the terms bound AICD and unbound AICD refer to these two trajectory subsets.

Residue–residue contact maps were calculated to quantify the intramolecular organization of unbound AICD and the mutant systems, as well as intermolecular recognition in the FE65–AICD complex. Pair distances, Ramachandran plots, and hydrogen bonds were analyzed using the GROMACS pairdist, rama,^28^ and hbond^20^ tools, respectively.

A pair of residues was defined as being in contact when the minimum distance between any pair of non-hydrogen atoms belonging to the two residues was less than or equal to 8 Å. Distances were calculated using the minimum-image convention under periodic boundary conditions. For unbound AICD and the mutant systems, contacts were calculated for all intramolecular residue pairs within the peptide. Residue pairs separated by fewer than |*i* – *j*| < 3 positions along the sequence were excluded from the analysis. For the FE65-bound system, only intermolecular contacts between AICD and FE65 residues were included.

The contact probability between residues *i* and *j* was calculated as

2 $$P_{\mathrm{ij}}=\frac{N_{\mathrm{ij}}}{N_{\mathrm{frames}}}$$

where *N*_*ij*_ is the number of analyzed frames in which residues *i* and *j* satisfied the contact criterion, and *N*_frames_ is the total number of analyzed frames. Contact probabilities were calculated from frames sampled every 100 ps after removal of the initial 300 ns equilibration period from each replica.

Contact probability maps were used to identify local contacts, long-range intramolecular contacts, and FE65–AICD interfacial contacts. The robustness of the contact patterns was assessed using alternative heavy-atom distance cutoffs of 8.0 and 12.0 Å. The main contact regions and residue-level hotspot rankings were retained across these cutoff values.

To identify residue-level contact hotspots, a cumulative contact score was calculated for each AICD residue

3 $$S_{i}=\sum_{j}P_{\mathrm{ij}}$$

where the summation was performed over all contact partners *j* of residue *i*. For unbound AICD and mutant systems, *j* represents other AICD residues included in the intramolecular contact analysis. For the FE65-bound system, *j* represents FE65 residues. Thus, *S*_*i*_ measures the overall participation of AICD residue *i* in the intramolecular or intermolecular contact network, respectively.

We emphasize that *S*_*i*_ is an equilibrium, ensemble-averaged structural quantity: it reflects the population-weighted frequency with which residue *i* participates in the contact network across the sampled unbound or bound trajectory frames. It does not report on the order, rate, or free-energy cost of contact formation along a binding pathway, and should therefore not be interpreted as a kinetic or transition-state descriptor analogous to an experimental ϕ-value. Rather, *S*_*i*_ is best understood as a static, structure-based indicator of which residues are consistently engaged in the intramolecular or interfacial contact network at equilibrium.

### Principal Component Analysis and Free-Energy Landscapes

Principal component analysis (PCA)^29^ was used to characterize the dominant collective motions sampled by unbound and bound AICD. The covariance matrix of positional fluctuations was constructed from the aligned trajectory using selected Cα atoms. Diagonalization of the covariance matrix yielded eigenvectors corresponding to the principal components (PC). Each trajectory frame was projected onto the first two principal components (PC1 and PC2) to obtain a reduced-dimensional representation of the sampled conformational space. Free-energy landscapes were constructed from the probability distribution of conformations projected onto PC1 and PC2. The relative free energy was calculated as

4 $$F\;\left(\mathrm{PC}1,\mathrm{PC}2\right)=-k_{B}T\;\ln P\;\left(\mathrm{PC}1,\mathrm{PC}2\right)$$

where *k*_B_ is the Boltzmann constant, *T* is the simulation temperature, and *P*(PC1, PC2) is the normalized probability density. The minimum free-energy value was set to zero. These landscapes were used to compare the breadth and organization of the free and FE65-bound AICD ensembles.

### Clustering of Bound-State Conformations

The FE65–AICD trajectory was clustered using the GROMOS^30^ algorithm based on pairwise backbone Root Mean Square Deviation (RMSD)^20^ after alignment of the FE65 backbone. RMSD cutoffs between 0.7–1.2 nm were examined. A cutoff of 0.9 nm was selected because it yielded five well-populated conformational clusters without fragmenting structurally similar states into numerous small clusters. Analyses performed with neighboring cutoff values recovered by the same principal conformational families and did not alter the qualitative interpretation of AICD orientation and surface engagement.

### Analysis of YENPTY-Motif Mutants

The three YENPTY-motif mutant systems were analyzed using the same structural descriptors as those used in the wild-type AICD simulations. The *R*_*g*_ ^20^ distributions were used to compare global compactness; secondary structure fractions were evaluated as a function of *R*_*g*_; and Cα contact maps were used to identify changes in the intramolecular contact network. Comparison of the N684A mutant, the ΔNPTY deletion mutant, and the multiresidue alanine mutant allowed the effect of local motif disruption on AICD ensemble organization to be assessed.

### Statistical Analysis and Convergence Assessment

All structural properties of the mutant AICD systems were calculated from the equilibrated portions of the production trajectories. Equilibration was assessed separately for each replica by monitoring the time evolution and running averages of the *R*_*g*_, secondary structure content, and total number of intramolecular residue–residue contacts. The first 300 ns of each 1 μs production trajectory were excluded from the analysis. The remaining 700 ns were used for ensemble characterization because the monitored observables fluctuated around stable mean values without a persistent drift over this interval.

Convergence was evaluated by comparing the distributions and running averages of these observables among the five independent replicas and among successive trajectory blocks. Each equilibrated 700 ns trajectory was divided into five nonoverlapping blocks of 140 ns. The principal features of the *R*_*g*_,^20^ secondary-structure, and intramolecular-contact distributions were reproduced across replicas and blocks, indicating that the reported mutant ensembles were not dominated by a single replica or trajectory segment.

Secondary structure convergence^20^ was assessed from the block-wise and replica-wise fractions of helix, β-structure, turn, and coil conformations. Similar secondary-structure populations across the analyzed blocks and replicas were taken as evidence that secondary-structure propensities were stable over the time scale analyzed. For scalar and distribution-based observables, values were calculated independently for each replica and trajectory block. The reported values correspond to the mean across the replica–block estimates, and the uncertainty is reported as the standard error of the mean,

$$\mathrm{SEM}=\frac{s}{\sqrt{N}}$$

where *s* is the standard deviation of the replica–block estimates and *N* = 25 is the total number of estimates. Probability distributions were normalized over the analyzed frames. The same trajectory-processing and analysis workflow was applied to all three mutant systems to ensure direct comparability.

### Nonlinear Clustering of Bound-State Conformations

As a complementary, nonlinear approach to the GROMOS RMSD-cutoff clustering described above, t-distributed stochastic neighbor embedding (t-SNE) combined with K-means clustering was performed on the FE65-bound AICD trajectory, following the framework of Appadurai et al.^31^ An all-to-all pairwise Cα-RMSD matrix was computed for the bound AICD conformations across both FE65–AICD complexes in the simulation box, using a local Kabsch superposition for each pair of conformations. t-SNE was then applied directly to this precomputed distance matrix (rather than to Cartesian coordinates) to obtain a two-dimensional embedding, and K-means clustering was performed on the resulting embedding.

For each combination of t-SNE perplexity and number of K-means clusters *K*, an integrated Silhouette score was calculated

5 $$S=S_{hd}\times S_{ld}$$

where *S*_hd_ is the Silhouette score computed using the original, high-dimensional pairwise-RMSD distances, and *S*_ld_ is the Silhouette score computed using Euclidean distances in the two-dimensional t-SNE embedding. This integrated score penalizes partitions that appear well separated only in the low-dimensional projection but not in the underlying RMSD-based distance metric, or vice versa. Perplexity values from 10 to 150 and cluster numbers *K* = 2 to 10 were scanned, and the combination that maximized the integrated Silhouette score (perplexity = 100, *K* = 3) was selected for the final clustering and is reported in Figure 7.

## Results and Discussion

### FE65 Binding Redistributes the AICD Conformational Ensemble

Figure 1 illustrates representative conformations of AICD in its unbound and FE65-bound states. In the unbound state (Figure 1a), AICD displays an extended conformation with local secondary structural elements connected by flexible regions. In the FE65-bound state (Figure 1b), AICD is positioned at the interaction interface of the compact PTB2 domain of FE65. These snapshots provide a structural overview of the two simulated systems and illustrate the spatial arrangement of AICD in the absence and presence of FE65. As individual conformations do not capture the full structural variability sampled during the simulations, differences between the unbound and bound states are examined through the subsequent quantitative analyses of the conformational ensembles.

**Figure 1:**
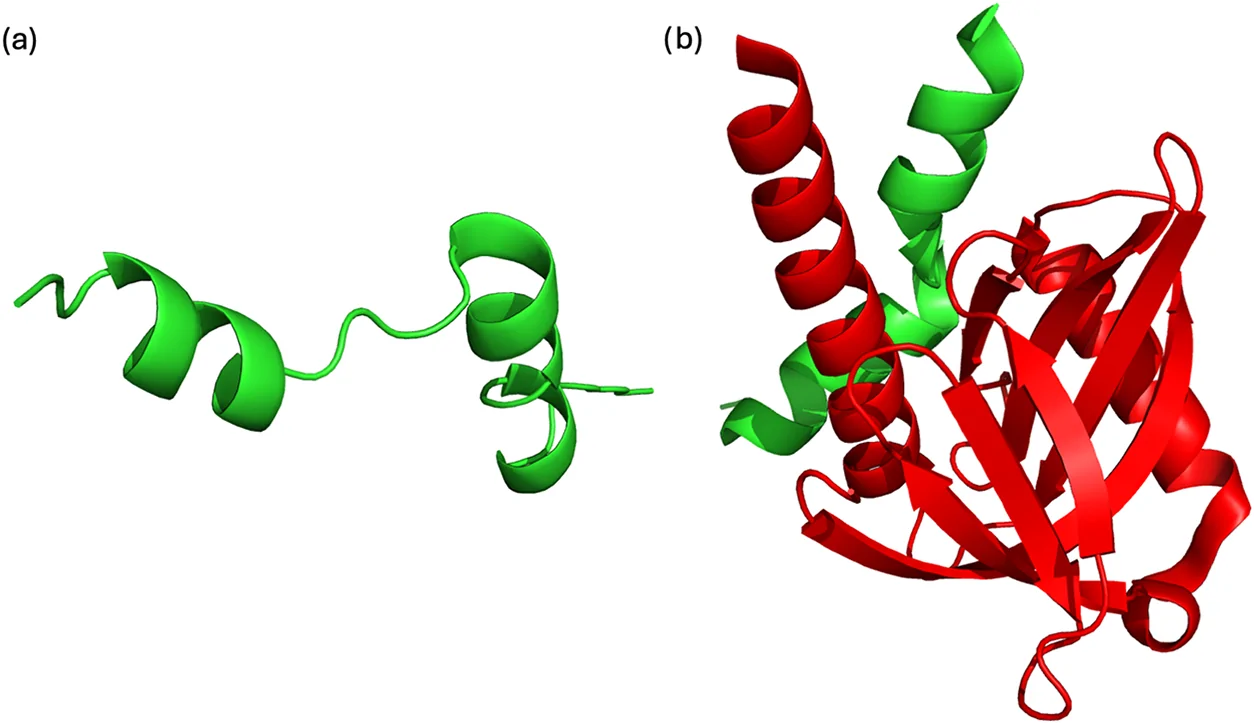
Representative conformations of AICD in (a) its unbound state and (b) complex with FE65. AICD is shown in green, while FE65 is depicted in red.

To compare the global dimensions sampled by the unbound and bound AICD, we calculated the probability distributions of the *R*_*g*_ (Figure 2). The left panel represents AICD in the unbound state and shows a relatively narrow, approximately unimodal distribution centered at ⟨*R*_*g*_⟩ = 15.20 Å, with a standard deviation of 0.75 Å. This distribution indicates that unbound AICD predominantly samples a moderately compact ensemble with comparatively limited fluctuations in its global dimensions.

**Figure 2:**
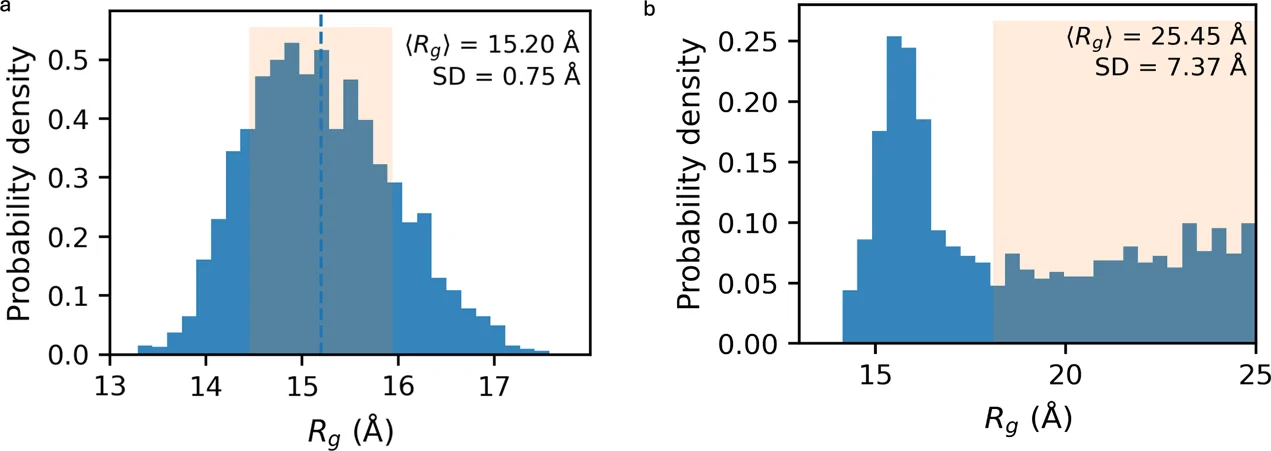
Probability density distributions of the radius of gyration (*R*_*g*_) for (a) unbound and (b) bound AICD. Thus, the absolute *R*_*g*_ values are not directly comparable as peptide-only compactness measures because FE65 contributes to the size of the bound system. Unbound AICD is centered at *R*_*g*_ = 15.20 Å with a standard deviation of 0.75 Å, indicating a moderately compact but heterogeneous ensemble. The FE65+AICD complex has a larger mean *R*_*g*_ = 25.45 Å and a broader distribution with a standard deviation of 7.37 Å, reflecting the wider range of global configurations sampled by the protein–peptide assembly.

In contrast, the bound AICD exhibits a substantially broader and strongly right-skewed *R*_*g*_ distribution, with ⟨*R*_*g*_⟩ = 25.45 Å and a standard deviation of 7.37 Å. A prominent population is observed near *R*_*g*_ ≈ 15–17 Å, followed by a broad distribution extending toward larger values, indicating that the bound AICD samples a wide range of global conformations. The larger mean and greater variability of the bound AICD reflect the dimensions and collective motions of the entire protein-peptide assembly. Because the two panels correspond to different structural selections, the unbound and bound AICD, their absolute *R*_*g*_ values should not be interpreted as a direct measure of binding-induced expansion or compaction of AICD alone.

The *R*_*g*_ analysis therefore supports two complementary conclusions. First, unbound AICD is heterogeneous but not fully expanded; instead, it occupies a moderately compact disordered ensemble. Second, the bound AICD remains globally dynamic and samples multiple large-scale arrangements. These observations are consistent with a recognition mechanism in which binding reshapes the accessible conformational landscape without freezing the system into a single rigid state.

The contact analysis reveals distinct organizational features for unbound and bound AICD (Figure 3). The unbound AICD intramolecular contact probability map (Figure 3a) shows a pronounced diagonal band, reflecting frequent local contacts between neighboring residues, along with several off-diagonal regions of elevated contact probability. These off-diagonal features indicate that unbound AICD is not a completely random coil, but instead samples recurrent conformations in which separated sequence regions transiently approach one another. Thus, the free peptide retains a degree of internal organization despite its overall disordered character.

**Figure 3:**
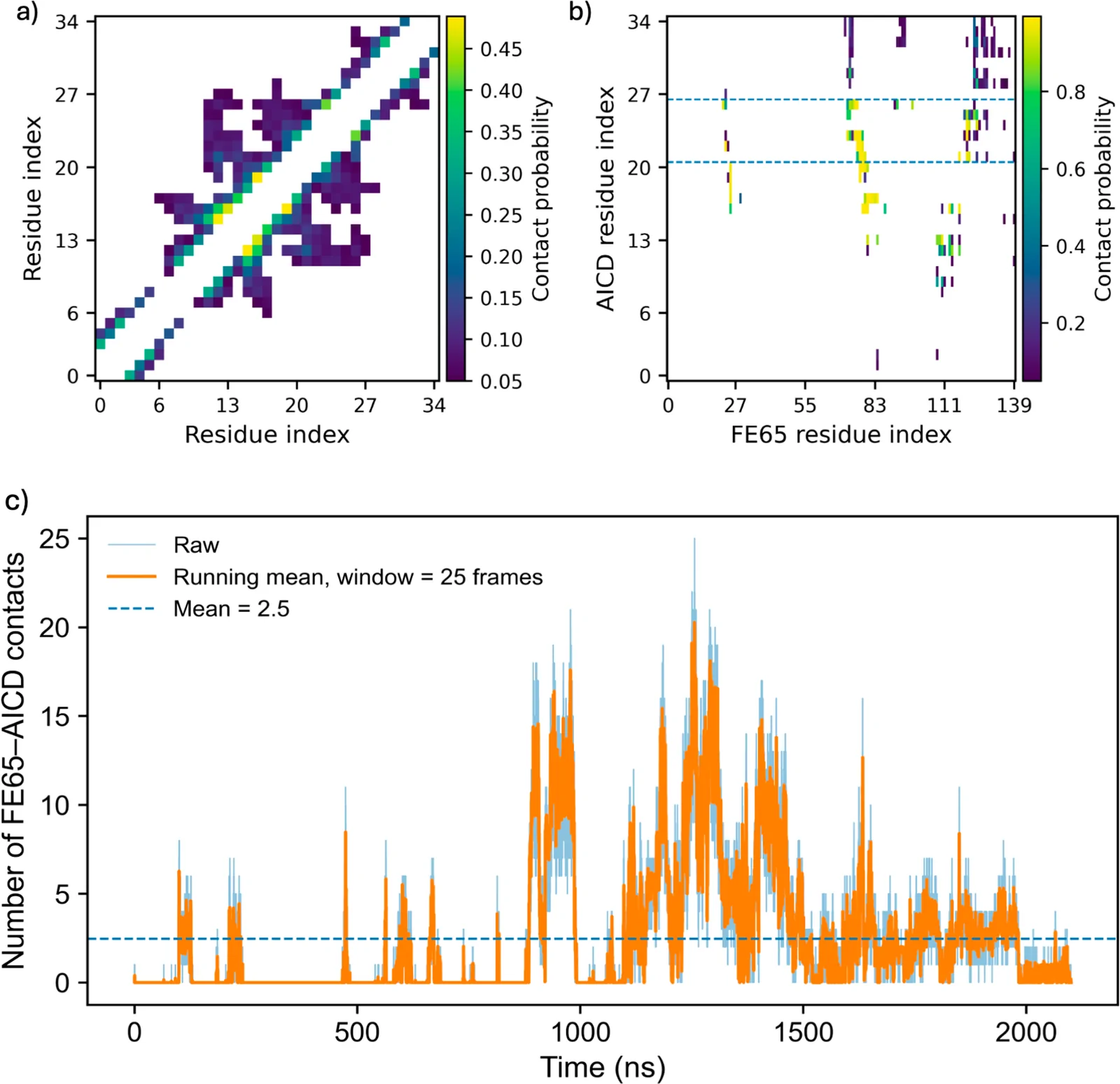
Contact organization and intermolecular contact dynamics of unbound and bound AICD. (a) Intramolecular contact probability map of unbound AICD, showing frequent local contacts along the diagonal together with recurrent longer-range contacts between non-neighboring residue regions. (b) Intermolecular FE65–AICD contact probability map of bound AICD. Rows correspond to AICD residue indices and columns correspond to FE65 residue indices. Dashed horizontal lines indicate the conserved YENPTY region. Contacts are concentrated within selected AICD and FE65 residue regions. (c) Time evolution of the total number of FE65–AICD residue–residue contacts. The raw contact count is shown together with a 25-frame running average and the trajectory average.

The intermolecular contact map of the FE65-AICD complex (Figure 3b) shows a much sparser pattern. Rather than forming a continuous interface across the full peptide, FE65 contacts are concentrated in discrete residue regions of AICD and FE65. The most populated contacts occur near the YENPTY-containing region of AICD, with additional contributions from neighboring AICD residues. On the FE65 side, the contacts are similarly localized to selected residue patches rather than being broadly distributed across the entire domain. This pattern is consistent with a hotspot-mediated recognition model, in which a limited number of preferred interaction regions contribute disproportionately to stabilizing the complex, rather than establishing a uniformly distributed binding interface.

The contact time series further shows that the FE65-AICD interface is dynamic (Figure 3c). The average number of intermolecular contacts is low, approximately 2.5 contacts over the trajectory, but the system undergoes intermittent bursts in which the contact number increases substantially. Although instantaneous contacts fluctuate strongly, the recurrence of contact-rich intervals supports the robustness of the observed interaction pattern. These bursts indicate transient formation of more highly connected bound substates, whereas periods of lower contact number reflect partial relaxation of the interface. The contact map and contact dynamics suggest that FE65 recognition of AICD is not governed by a single rigid binding geometry. Instead, the complex appears to be maintained by a fluctuating ensemble of localized, recurring contacts, consistent with dynamic recognition of an intrinsically disordered peptide segment.

### Ensemble Redistribution Revealed by PCA

The PCA-based free energy landscapes reveal clear differences between the conformational sampling of unbound and bound AICD (Figure 4). For unbound AICD (Figure 4a), the first two PCs account for 38.2% of the total variance, indicating that the dominant motions are distributed over multiple conformational degrees of freedom. The corresponding free energy landscape is broad and rugged, with several low-energy regions distributed across the PC1–PC2 space. This behavior is consistent with an intrinsically disordered peptide that samples multiple interconverting conformational substates rather than a single dominant structure.

**Figure 4:**
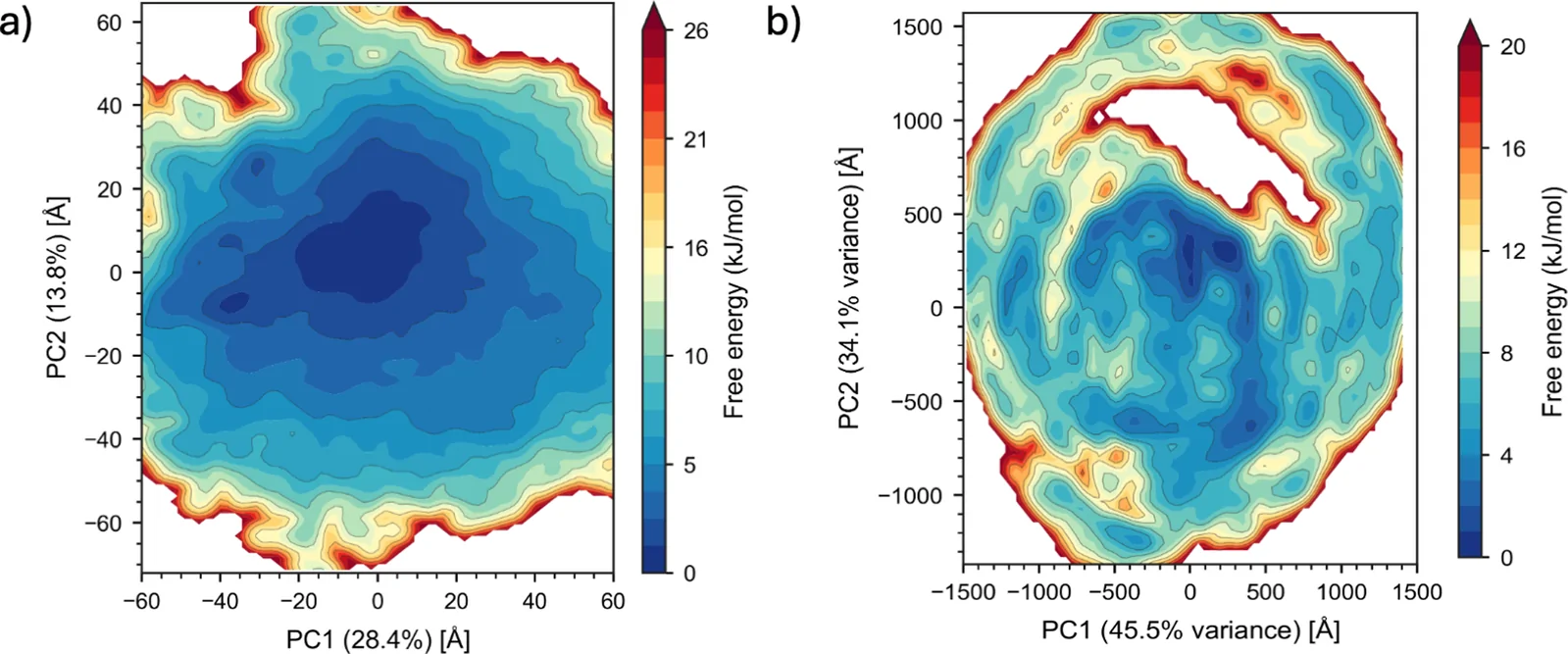
Principal component analysis-based free-energy landscapes of unbound and bound AICD projected onto the first two principal components (PC1 and PC2). (a) Unbound AICD exhibits a broad and rugged free-energy landscape with multiple low-energy regions. (b) Bound AICD exhibits a more localized free-energy landscape with low-energy regions concentrated within a narrower region of PC space.

For the bound AICD (Figure 4b), the first two PCs account for a substantially larger fraction of the total variance, 71.6%, indicating that the dominant motions of bound AICD are more strongly captured by the leading collective coordinates. The free energy landscape remains heterogeneous but appears more organized, with low-energy regions concentrated within a more defined region of PC space and higher-energy states located toward the periphery. Because these landscapes are projected onto PC1 and PC2, they should be interpreted as reduced-dimensional representations of the conformational ensemble rather than as complete free-energy surfaces. These landscapes indicate that FE65 binding redistributes the AICD conformational ensemble toward preferred substates while preserving substantial conformational heterogeneity.

The projection in Figure 5 provides a visual assessment of the clustering of the sampled configurational space. In the PC1–PC2 representation shown in Figure 5a, the clusters occupy a relatively compact region with considerable overlap, indicating that the first two linear modes capture important variance but do not fully separate all configurational substates. Nevertheless, the nonuniform spatial distribution of the colored points suggests the presence of partially resolved subpopulations. In Figure 5b, the same cluster assignments are shown in a low-dimensional representation that separates the data into more clearly defined regions, supporting the presence of multiple structurally or dynamically distinguishable substates within the sampled ensemble.

**Figure 5:**
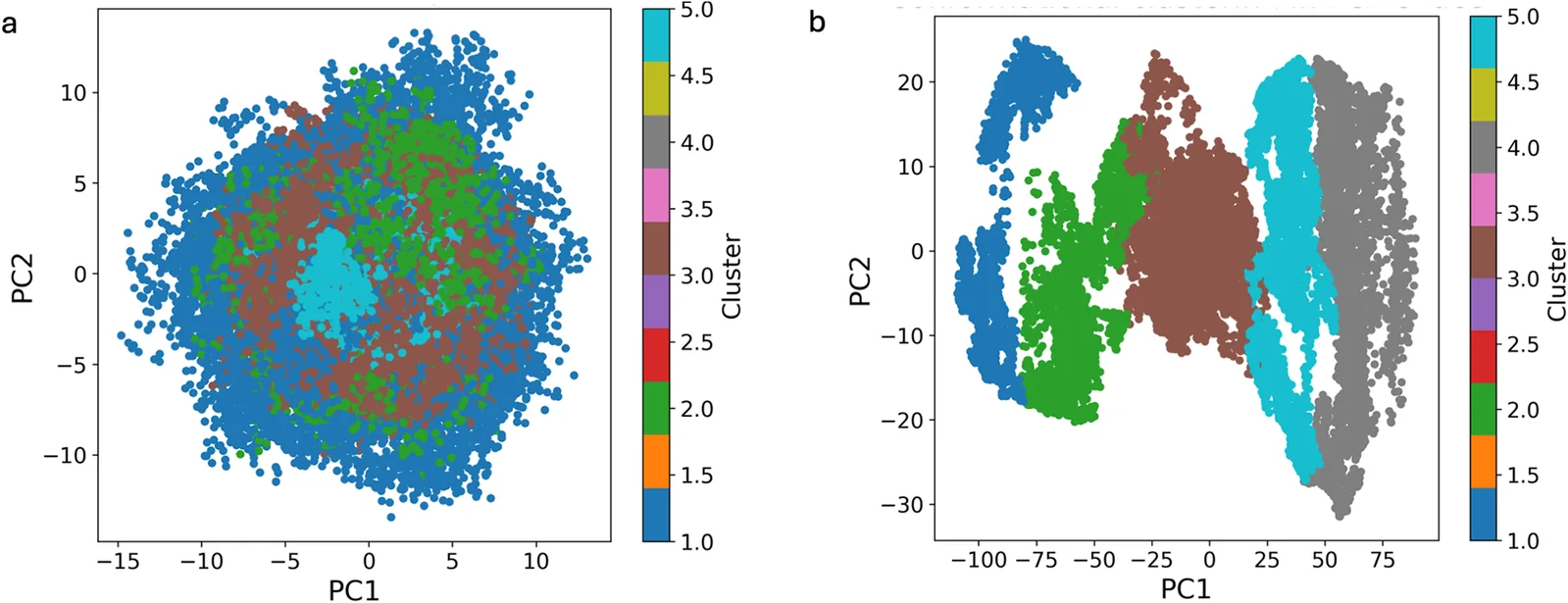
Cluster assignments of the FE65-AICD bound-state trajectory projected in reduced-dimensional space. (a) Frames colored by cluster assignment, projected onto PC1 and PC2, showing a compact region with substantial overlap between clusters. (b) The same cluster assignments shown in an alternative low-dimensional projection that more clearly separates the five conformational substates.

Five clusters provided a practical description of the dominant conformational substates without oversubdividing closely related structures. Cluster 3 was the most populated, followed by clusters 4 and 5, whereas clusters 1 and 2 were less populated. The results indicate that the FE65-AICD complex occupies multiple bound substates rather than a single rigid configuration.

Representative clustered conformations of the FE65–AICD complex (Figure 6) reveal substantial heterogeneity in the bound ensemble. The corresponding cluster populations are reported in Supporting Figure S6 and show that the five representative states are not equally populated. Cluster 3 is the dominant substate, containing approximately 6731 frames, followed by clusters 4 and 5 with approximately 4515 and 4323 frames, respectively. Clusters 2 and 1 are less populated, with approximately 3254 and 2138 frames, respectively. Thus, all five clusters contribute to the sampled ensemble, but the distribution is biased toward a subset of more frequently visited bound conformations.

**Figure 6:**
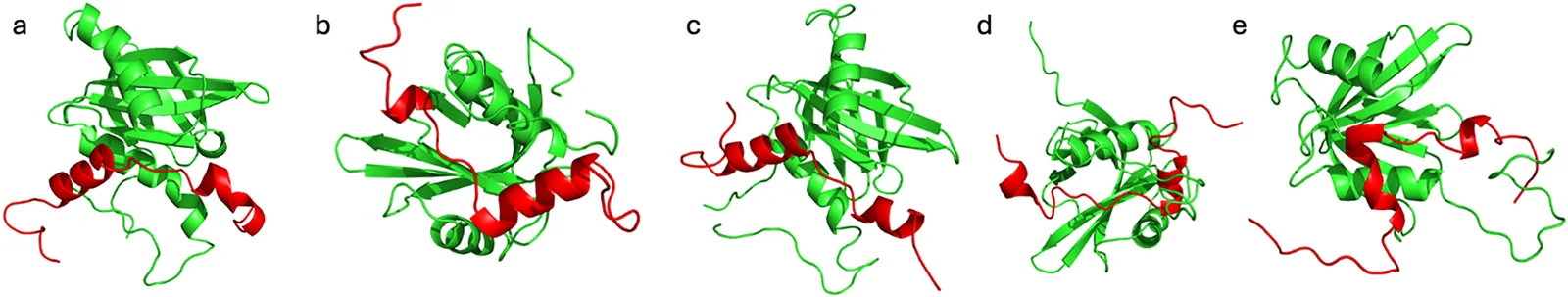
Representative structures of the FE65–AICD complex obtained from the five conformational clusters identified in the bound-state trajectory. Panels (a)–(e) correspond to clusters 1–5, respectively. FE65 is shown in green and AICD in red. The representative structures illustrate distinct orientations and degrees of surface engagement of bound AICD at the FE65 interface.

FE65, shown in green, retains a compact folded architecture across all clusters, whereas AICD, shown in red, samples multiple binding geometries on the FE65 surface. In each representative structure, AICD remains partly structured, with short helical segments connected by flexible or extended regions, indicating that binding does not fully rigidify the peptide. Instead, AICD appears to engage FE65 through a combination of localized structured contacts and conformationally variable flanking regions.

The representative structures highlight the dynamic nature of the FE65-AICD interaction. Although AICD remains associated with FE65 in all clusters, its orientation and degree of surface engagement vary considerably, indicating that the complex is better described as an ensemble of related bound conformations than as a single rigid binding mode. This structural variability is consistent with the residue-level hotspot analysis.

A complementary nonlinear clustering analysis (t-SNE combined with K-means; Figure 7) yields a qualitatively consistent picture, with only partial cluster separability (integrated Silhouette score = 0.08), further supporting a model of continuously interconverting bound substates rather than discretely resolved binding conformations.

**Figure 7:**
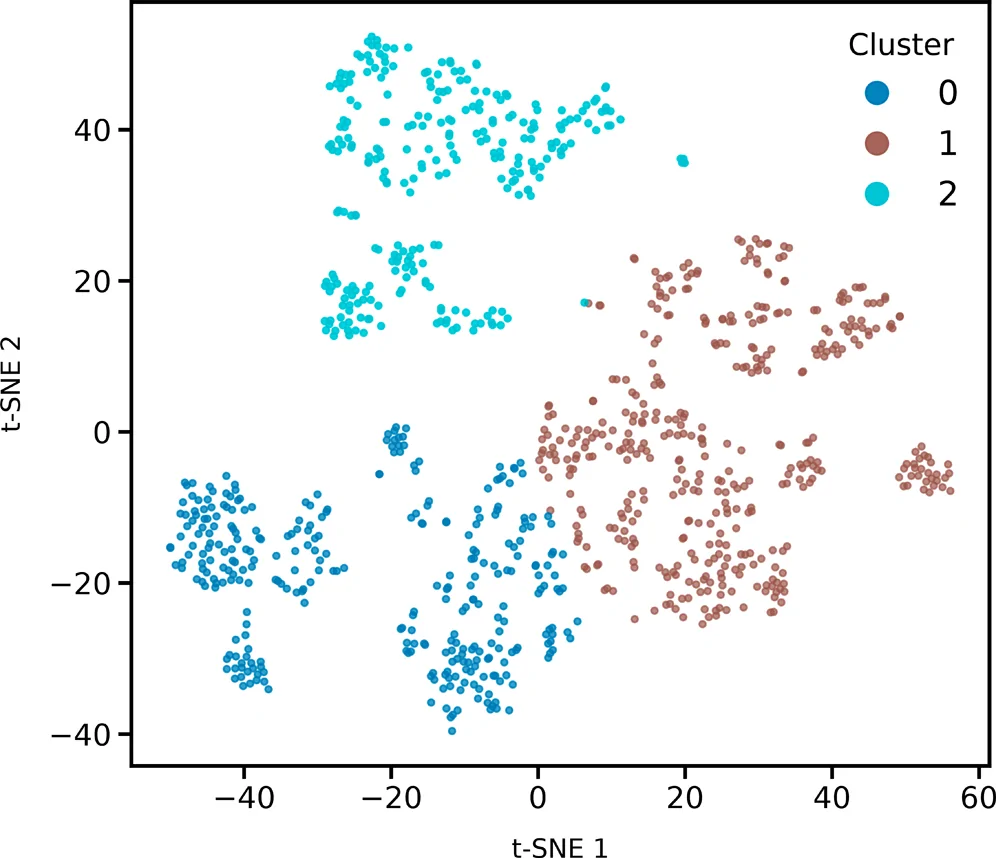
t-distributed stochastic neighbor embedding (t-SNE) projection of bound AICD conformations clustered using K-means. Pairwise Cα-RMSD distances between bound AICD conformations (FE65–AICD complex in the simulation box) were embedded in two dimensions using t-SNE applied directly to the precomputed RMSD matrix. The perplexity (100) and number of K-means clusters (K = 3) shown were selected as the combination maximizing the integrated Silhouette score (S = Shd × Sld), defined as the product of the Silhouette scores computed from the high-dimensional pairwise RMSD distances and the low-dimensional t-SNE embedding (see Methods). Points are colored according to their K-means cluster assignments (clusters 0, 1, and 2). The integrated Silhouette score of 0.08 indicates limited overall cluster separability, suggesting that the bound conformations are not sharply separated into distinct structural clusters.

### Binding-Induced Reorganization of AICD Residue Contact Networks

To evaluate whether the residue-level hotspot rankings were stable across the trajectory, the trajectory was divided into five equal blocks, and the cumulative contact-score ranking was recalculated independently for each block. The leading residues showed strong robustness in ranking (Table 1). In particular, Y16 remained the top-ranked residue in every block, with a mean block rank of 1.0 and no rank variation. Y21, Q1, N18, M11, Q12, T20, and F24 were also recovered within the top 10 in all five blocks, indicating that the dominant hotspot pattern is not driven by a single trajectory segment. H2, L8, P19, and M27 showed lower recurrence, appearing in the top 10 in three, two, three, and two of the five blocks, respectively. In contrast, residues ranked below the top 10 generally showed reduced or absent top-10 recurrence and greater rank variability, suggesting that the most robust contact hotspots are concentrated within a limited subset of AICD interfacial residues.

**Table 1:** Block-Wise Robustness of Residue-Level Contact-Score Rankings for the Selected AICD Chain^a^,^b^

Residue	Full score (× 10^6^)	Full rank	Mean block rank	SD block rank	Top-10 recurrence
Y16	1.609	1	1.0	0.00	5/5
Y21	1.066	2	2.2	0.45	5/5
Q1	1.001	3	4.8	2.17	5/5
N18	0.938	4	3.8	0.84	5/5
M11	0.917	5	4.8	0.84	5/5
Q12	0.896	6	4.8	1.30	5/5
T20	0.691	7	7.0	1.22	5/5
F24	0.674	8	7.6	0.55	5/5
H2	0.460	9	10.8	2.17	3/5
L8	0.444	10	11.2	1.92	2/5
P19	0.440	11	11.0	2.55	3/5
E17	0.350	12	12.8	1.10	0/5
*F*23	0.339	13	14.0	2.74	0/5
M27	0.338	14	12.8	5.17	2/5
G15	0.327	15	14.2	1.30	0/5
N14	0.211	16	16.6	0.55	0/5
H3	0.210	17	17.0	2.74	0/5
H4	0.139	18	18.8	2.17	0/5
H5	0.121	19	19.4	4.04	0/5
Q13	0.106	20	19.6	1.82	0/5

a Residue numbering is relative to the selected AICD segment. Top-10 recurrence indicates the number of trajectory blocks in which a residue appeared among the ten highest-ranked residues.

b Group B residues are numbered relative to the selected AICD segment. The trajectory was divided into five equal blocks, and contact-score rankings were recalculated independently for each block. Full contact scores are reported in units of 10^6^.

The cumulative contact-score analysis further reveals a marked reorganization of AICD residue-level interactions upon FE65 binding. In the unbound state, the highest-ranked residues exhibit relatively modest, narrowly distributed contact scores, with Q17, L13, and M16 contributing most strongly (Table 2). These residues, together with Q18, H12, K27, S14, and P24, define the principal intrinsic contact-prone region of AICD in the absence of binding. The limited score range in the unbound ensemble suggests that contacts are comparatively weak and dispersed, consistent with a conformationally flexible state in which no single residue dominates the interaction network.

**Table 2:** Top-Ranked AICD Residues in the Unbound State Based on Cumulative Contact Score

Rank	Residue	Cumulative contact score
1	Q17	2.72
2	L13	2.58
3	M16	2.38
4	Q18	2.17
5	H12	1.88
6	K27	1.86
7	S14	1.78
8	P24	1.71

In contrast, the bound state exhibits substantially larger cumulative contact scores, indicating pronounced stabilization and strengthening of residue-specific contacts upon complex formation (Table 3). The dominant contributors in the bound ensemble are Y26 and Y21, followed closely by M16, Q17, and N23. When these residues are mapped onto the FE65–AICD structural model, they localize to the AICD segment positioned at the FE65 PTB2 binding interface, including residues within and adjacent to the YENPTY-containing region. This spatial correspondence links the quantitative contact-score analysis directly to the structural model and supports the interpretation of these residues as interface-associated hotspots rather than nonspecific high-contact positions.

**Table 3:** Top-Ranked AICD Residues in the Bound State Based on Cumulative Contact Score

Rank	Residue	Cumulative contact score
1	Y26	7.761246
2	Y21	7.256581
3	M16	7.220427
4	Q17	7.141953
5	N23	7.101300
6	L13	5.442353
7	T25	5.240920
8	G20	4.871043
9	H12	4.297901
10	E22	4.165445

The emergence of Y26 and Y21 as the two highest-ranked bound-state residues suggests that aromatic side chains make an important contribution to the FE65-bound interaction network. This observation is consistent with known structural features of PTB-domain recognition, in which NPxY/YENPTY-containing motifs are recognized through a combination of motif-specific anchoring and surrounding hydrophobic, aromatic, polar, and packing interactions. In this context, the aromatic residues may help stabilize the FE65 PTB2 interface through hydrophobic packing and possible π-associated contacts, while neighboring residues such as M16, Q17, and N23 provide additional stabilizing interactions across the extended interface. Thus, FE65 recognition appears to involve a broader contact surface than the canonical YENPTY motif alone.

Notably, M16 and Q17 remain highly ranked in both the unbound and bound states. This persistence suggests that these residues may act as preorganized contact-prone sites in the unbound AICD that become further stabilized upon FE65 binding. Therefore, a comparison of the two states indicates that binding does not simply amplify the pre-existing unbound contact pattern. Instead, FE65 binding redistributes the dominant contacts toward the central-to-C-terminal region of AICD, particularly the Y21–Y26 segment. Residues such as L13 and H12 retain appreciable contact contributions in the bound state, although their relative ranks decrease, whereas residues including K27, S14, and P24 are prominent mainly in the unbound top-ranked set. Together, these findings support a model in which FE65 binding reorganizes a weakly distributed intrinsic AICD contact network into a more focused hotspot cluster at the PTB2 binding interface.

The contact-segment length distributions reveal two distinct populations across the four simulation intervals: short localized contact segments and longer contiguous contact segments at the FE65–AICD interface (Figure 8). During the first interval (0.0–525.0 ns; Figure 8a), short segments comprising approximately 1–4 residues exhibit appreciable probabilities, with the 8.0 Å cutoff showing a pronounced peak at *N* ∼ 3. A second population is observed at larger segment lengths, particularly between *N* ∼ 23 and 30. For the 8.0 Å cutoff, a distinct peak occurs near *N* ∼ 23, whereas the 10.0 Å cutoff exhibits its maximum near *N* ∼ 28. The 12.0 Å profile displays a broader distribution around N ∼ 28–29. During the second interval (525.1–1050.0 ns; Figure 8b), a similar bimodal pattern persists, although the probabilities of the longer segments are generally lower than those observed in the first interval. The 8.0 Å profile retains prominent features near *N* ∼ 23 and 27, while the 10.0 and 12.0 Å profiles show peaks around *N* ∼ 28–29. In the third interval (1050.1–1575.0 ns; Figure 8c), the distributions become broader and less sharply defined, with a reduction in the maximum probabilities of both short and extended contact segments. Nevertheless, distinct populations remain evident near *N* ∼ 23–24 and *N* ∼ 28–30. During the final interval (1575.1–2100.0 ns; Figure 8d), short contact segments continue to be sampled, while longer segments exhibit relatively broad distributions with moderate probabilities around *N* ∼ 23–29. Notably, segments approaching the full 35-residue length of AICD are also observed, particularly at the 12.0 Å cutoff, although their probabilities remain comparatively low. Across all four intervals, increasing the contact cutoff generally shifts the prominent long-segment population toward larger *N* values, consistent with the inclusion of additional neighboring residues within the contact criterion. Overall, these results demonstrate the coexistence of localized and extended contiguous contact arrangements at the FE65–AICD interface throughout the 2.1 μs simulation. The temporal variations in their relative probabilities suggest a heterogeneous and dynamically evolving interface, in which short contact patches and extended interaction arrangements are sampled with different frequencies depending on the simulation interval and distance cutoff. Importantly, the presence of extended contact segments does not necessarily imply a rigid or continuously maintained interface but instead reflects the transient formation of contiguous interfacial contacts within the conformational ensemble.

**Figure 8:**
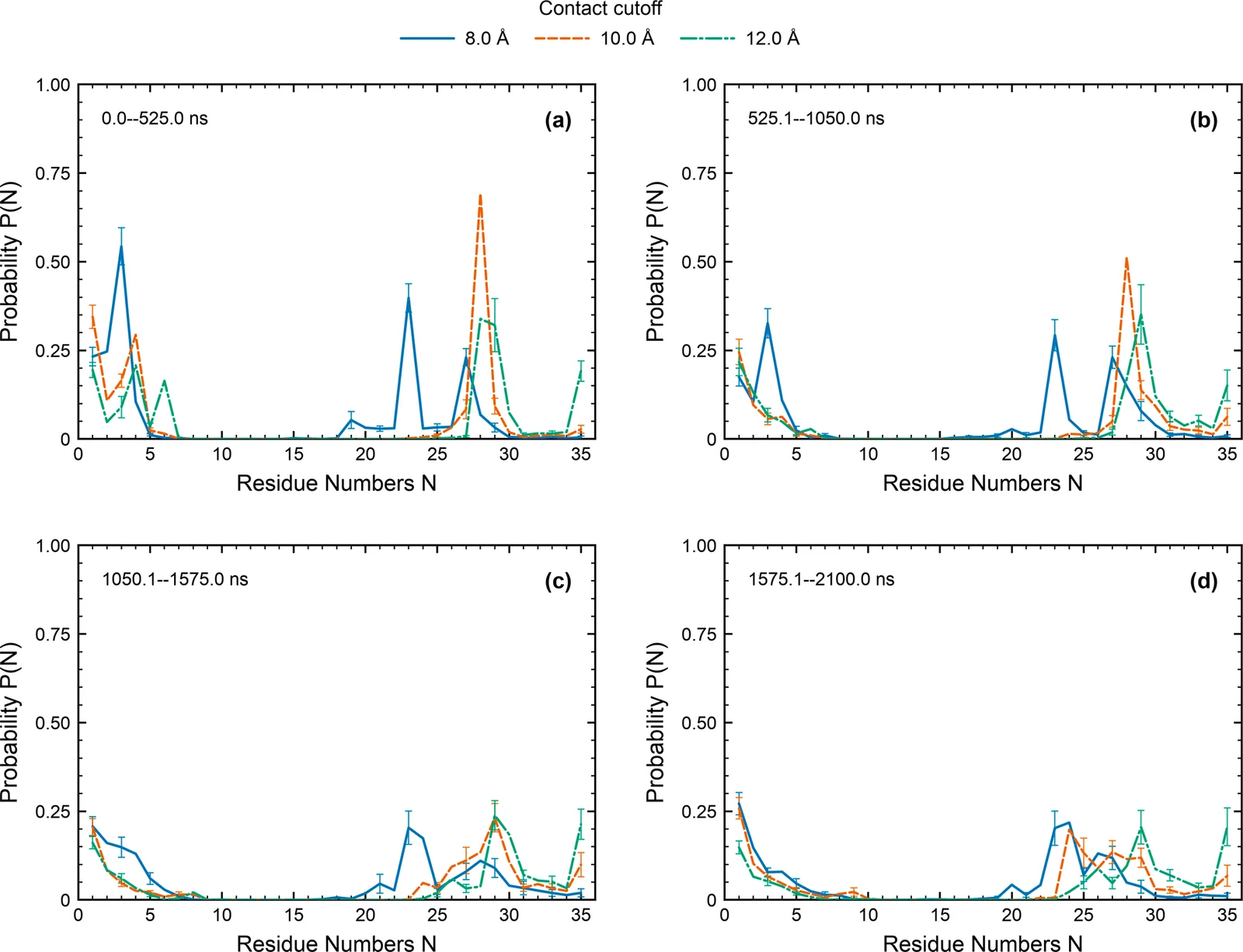
Probability, *P*(*N*), of observing at least one contiguous AICD residues *N* was calculated over four consecutive simulation intervals (a–d). Solid, dashed, and dash-dotted lines correspond to contact cutoffs of 8.0 (blue), 10.0 (orange), and 12.0 (green) Å, respectively. Data were averaged over two FE65–AICD copies and trajectory blocks. Error bars represent the standard error of the mean.

Overall, the results indicate that the FE65–AICD interface dynamically alternates between short localized contact patches and extended contiguous contact states. The persistence of both populations across all four time windows suggests that these interaction modes are repeatedly sampled over the full 2.1 μs trajectory, while their relative probabilities and preferred segment lengths vary with time and contact cutoff.

### Design of YENPTY-Motif Mutants

AICD contains an intact YENPTY motif, highlighted in the boxed region of Figure 9. To examine the contribution of individual residues within this motif, three mutant constructs were designed. Mutant 1 introduces a single amino acid substitution in which Asn (N) is replaced by Ala (A). Mutant 2 contains a deletion of the NPTY segment within the motif. Mutant 3 represents an alanine-substitution mutant in which selected YENPTY residues are replaced by alanine while the surrounding sequence context is retained. The color-coded residue blocks facilitate visual comparison of conserved and altered amino acids among AICD and mutant sequences.

**Figure 9:**
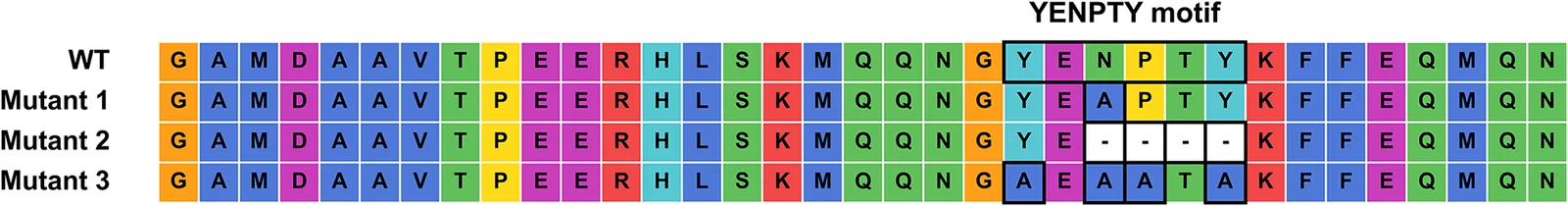
Sequence representation of the wild-type AICD and YENPTY-motif mutant constructs. The conserved YENPTY motif in the wild-type sequence is highlighted by the black box. Mutant 1 contains an N-to-A substitution, Mutant 2 a deletion of the NPTY segment, and Mutant 3 selected alanine substitutions within the motif. Colored blocks represent individual amino acid residues, and black outlines indicate residues modified relative to the wild-type sequence.

### Conformational Compactness and Secondary-Structure Dependence of YENPTY-Motif Mutants

*R*_*g*_ was analyzed to evaluate the compactness and conformational heterogeneity of the three mutant systems. As shown in Figure 10, Mutant 1 predominantly samples lower *R*_*g*_ values, indicating a relatively compact conformational ensemble. In contrast, Mutant 2 exhibits the largest mean *R*_*g*_ and a broad distribution, suggesting enhanced structural expansion and increased conformational variability. However, because Mutant 2 differs from the other systems in both sequence length and sequence composition, its increased *R*_*g*_ should be interpreted with caution. The observed expansion may reflect the combined effects of deletion-induced changes in chain properties and disruption of the YENPTY-mediated interaction network, rather than a single structural mechanism. Mutant 3 also displays a wide *R*_*g*_ distribution, with a mean value intermediate between Mutant 1 and Mutant 2, indicating that this system samples both compact and extended conformations. The extended right-hand tails observed for Mutant 2 and Mutant 3 further suggest transient sampling of highly expanded conformational states during the simulation.

**Figure 10:**
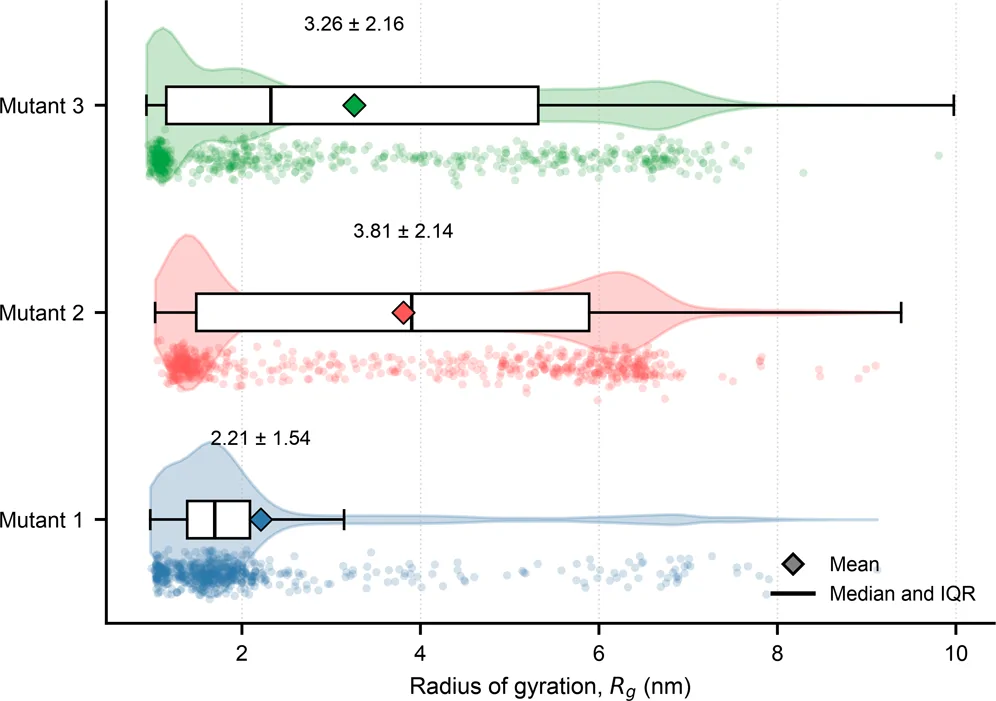
Distribution of the radius of gyration (*R*_*g*_) for Mutants 1–3. The raincloud plots combine violin density plots, individual sampled conformations, and box plots to illustrate the conformational distributions of the three mutant systems. The white box indicates the median and interquartile range, and the diamond denotes the mean. Reported values correspond to the mean ± standard deviation.

To place these compactness changes in the context of the unbound WT ensemble, we compared each mutant’s mean *R_g_* to the WT value of *R*_*g*_ = 1.520 nm (SD = 0.075 nm). Mutant 1 shows the smallest increase in mean *R*_*g*_ relative to WT and remains substantially more compact than Mutants 2 and 3. Mutant 2 (*R*_*g*_ = 3.81 nm) and Mutant 3 (*R*_*g*_ = 3.26 nm) show a substantial increase in mean Rg relative to WT, indicating that deletion or multiresidue substitution within the YENPTY motif measurably expands the conformational ensemble beyond the range sampled by the unmodified peptide. We also compared the contact-score rankings of Table 2 (WT unbound hotspots) against the corresponding residues in each mutant. Residues Q17 and M16, among the top three WT contact-prone positions, retain elevated local contact scores in Mutant 1, whereas their contact contributions are diminished in Mutant 2 and Mutant 3, consistent with the broader loss of the local contact network accompanying motif disruption in these two variants.

The relationship between conformational expansion and secondary-structure content was further examined by calculating secondary-structure fractions as a function of *R*_*g*_ (Figure 11). For all three mutants, coil conformations dominate across nearly the entire sampled *R*_*g*_ range, indicating that the mutant ensembles remain largely disordered. Mutant 1 shows only minimal helical content and negligible β-sheet contribution, consistent with a compact but predominantly coil-rich ensemble. Mutants 2 and 3 show modest increases in helix and β-sheet fractions at selected *R*_*g*_ values, particularly in the low-to-intermediate *R*_*g*_ regions. However, these ordered contributions remain minor relative to the coil fraction. This result suggests that the mutations alter the overall ensemble dimensions more strongly than they change intrinsic secondary-structure propensity. Thus, the expanded conformations observed for Mutants 2 and 3 are likely driven primarily by disordered-chain extension and altered conformational sampling, rather than by the formation of stable secondary-structure elements.

**Figure 11:**
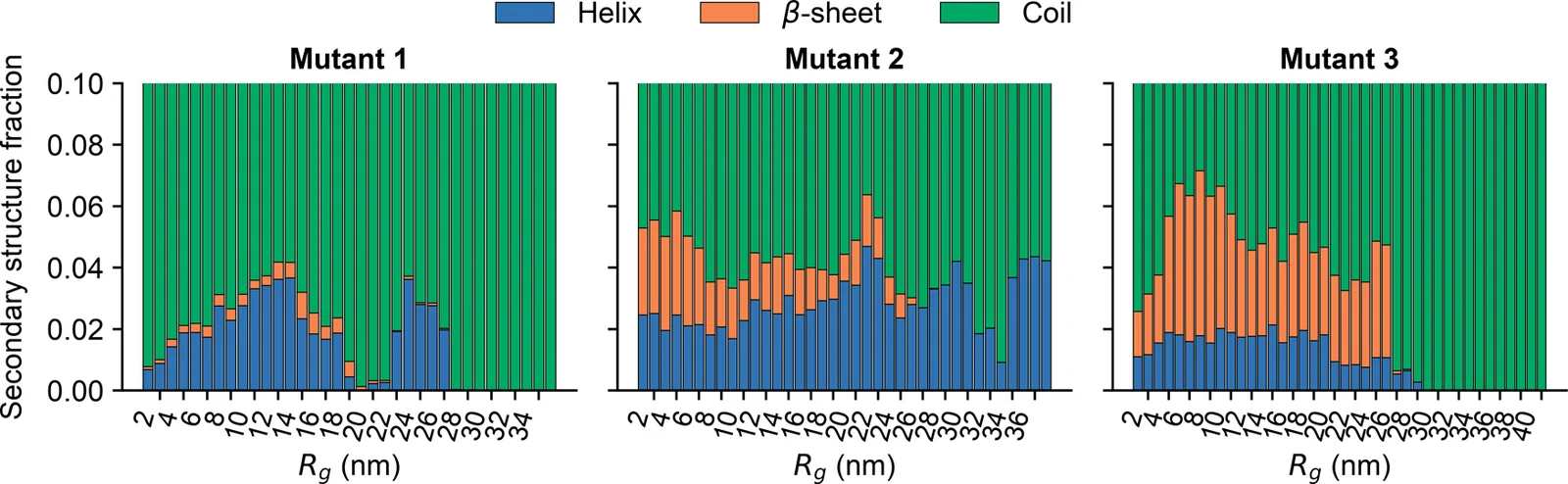
Secondary-structure fractions as a function of the radius of gyration (*R*_*g*_) for the three YENPTY-motif mutants. Stacked bars show the fractional populations of helix, β-sheet, and coil conformations within each *R*_*g*_ bin.

The contact maps (Figure 12) reveal that all three mutants retain prominent near-diagonal contacts, reflecting persistent local interactions between residues close in sequence. In addition to these local contacts, several off-diagonal contact regions are observed, indicating longer-range residue–residue interactions that contribute to the folded conformational ensemble. Mutant 1 shows relatively dispersed contact features with moderate off-diagonal interactions, suggesting a heterogeneous interaction network. Mutant 2 exhibits several stronger localized contact regions, including pronounced contacts around the midsequence residues, indicating enhanced stabilization of specific residue pairs. Mutant 3 displays a comparatively well-defined pattern of both local and nonlocal contacts, with notable contact enrichment in selected regions of the map.

**Figure 12:**
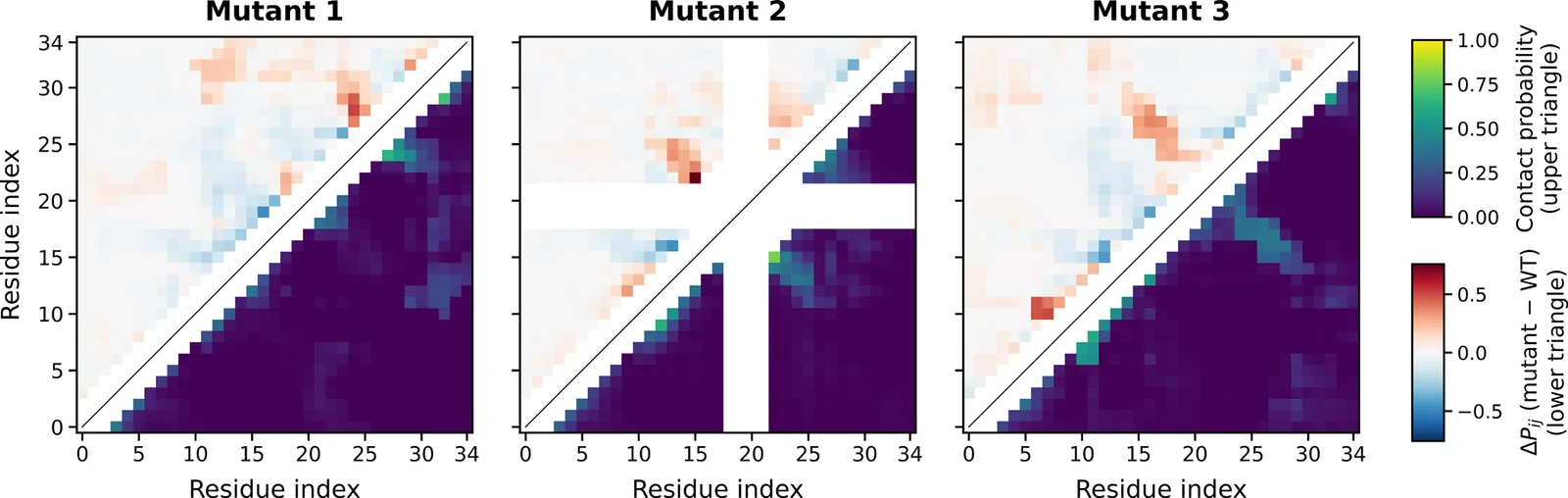
Residue–residue Cα contact probability maps for the three YENPTY-motif mutants, compared against the wild-type (WT) unbound ensemble. For each mutant, the upper triangle shows the mutant’s own contact probability map (viridis scale, 0–1), and the lower triangle shows the difference relative to WT, Δ*P*_*ij*_ = *P*_*ij*_(mutant) – *P*_*ij*_(WT), on a diverging red–blue scale centered at zero (red: contact gained relative to WT; blue: contact lost relative to WT). Residue indices follow WT numbering throughout. For Mutant 2 (the ΔNPTY deletion), residue pairs with no WT counterpart (i.e., involving the four deleted positions) are masked and shown in white. The three panels correspond to Mutant 1, Mutant 2, and Mutant 3, respectively.

## Conclusions

This study provides an ensemble-level description of AICD recognition by FE65 using MD simulations of unbound and bound AICD, and YENPTY-motif mutants. Unbound AICD populates a heterogeneous, intrinsically disordered ensemble with transient local structure and recurrent intramolecular contacts rather than behaving as a fully random coil. FE65 binding redistributes this ensemble toward interaction-compatible conformations while preserving substantial residual dynamics, supporting a recognition mechanism based on ensemble reweighting rather than complete disorder-to-order folding. We therefore propose dynamic hotspot-network reorganization as a useful description of AICD–FE65 recognition, in which binding is achieved not by folding into a single rigid structure, but by a shift in the relative weights of a fluctuating, localized contact network centered on the YENPTY region and its flanking residues.

Residue-resolved contact analysis identifies interaction hotspots within and adjacent to the YENPTY region, indicating that FE65 recognition is mediated by an extended binding interface rather than by the canonical motif alone. The YENPTY mutants further demonstrate that local sequence perturbations alter the balance between compact and extended conformations without inducing stable folding, suggesting that motif integrity influences the population of binding-competent states.

It is worth noting that the contact-score and hotspot rankings reported here are equilibrium, structural measures derived from simulation and are conceptually distinct from ϕ-value analysis, the standard experimental approach for probing transition-state structure in folding and binding kinetics. Because AICD-FE65 recognition involves a disordered, likely multipathway coupled folding-and-binding process rather than a classical two-state transition, direct comparison with ϕ-values would require dedicated kinetic measurements and pathway-resolved computational analysis. Future studies combining site-directed mutagenesis of the identified hotspot residues with kinetic binding assays would provide a valuable experimental complement to the present equilibrium, ensemble-based description.^32^

These results support a model in which AICD retains conformational flexibility while achieving specific FE65 recognition through localized interaction hotspots and redistribution of a dynamic conformational ensemble. Future work combining enhanced sampling, binding free-energy calculations, and experimental validation will help establish the energetic contributions of the identified hotspot residues and clarify how post-translational modifications regulate AICD–FE65 recognition. Given that the conformational ensembles of intrinsically disordered proteins are known to be force-field dependent, repeating key comparisons, particularly the unbound Rg distribution and secondary-structure propensities, using the a99SB-disp force field,^32^ which was specifically reparameterized for intrinsically disordered proteins, would provide a valuable independent check on the robustness of the ensemble-reweighting and hotspot-mediated recognition mechanism proposed here.

## Supplementary Material



## Notes

Additional analyses supporting the conformational and interaction characterization of the AICD and FE65–AICD systems; these data include backbone RMSD profiles; time-dependent intramolecular and intermolecular contact analyses; residue-wise cumulative contact scores for AICD and FE65, secondary-structure population changes upon FE65 binding, residue-resolved AICD contact-hotspot profiles; and the population distributions of the major conformational clusters identified for the FE65–AICD complex (PDF)

## Abbreviations

APP: amyloid precursor protein
AICD: amyloid precursor protein intracellular domain
AD: Alzheimer’s disease
FE65: APP adaptor protein FE65
PTB: phosphotyrosine-binding
IDP: intrinsically disordered protein
MD: molecular dynamics
RMSF: root-mean-square fluctuation
PCA: principal component analysis
*R*_*g*_: Radius of gyration
