# Supplementary material for: FE65 Recognition Rewires the Dynamic Contact Network of the Amyloid Precursor Protein Intracellular Domain
Source: J Phys Chem B. 2026 Oct 23;130(44):11718–31. doi: 10.1021/acs.jpcb.6c04695 (PMC13746784; doi:10.1021/acs.jpcb.6c04695)
Supplement: Supplementary file 1 [file jp6c04695_si_001.pdf]

FE65 Recognition Rewires the Dynamic Contact Network of the Amyloid Precursor Protein Intracellular Domain

Nabanita Mandal[1,2] and Marie Skepö*[1,2]

[1]Division of Computational Chemistry, Department of Chemistry, Science for Life Laboratory, Lund University, P.O. Box 124, Lund, Sweden
[2]NanoLund, Lund University, Box 118, Lund, Sweden

*Email: marie.skepo@chem.lu.se

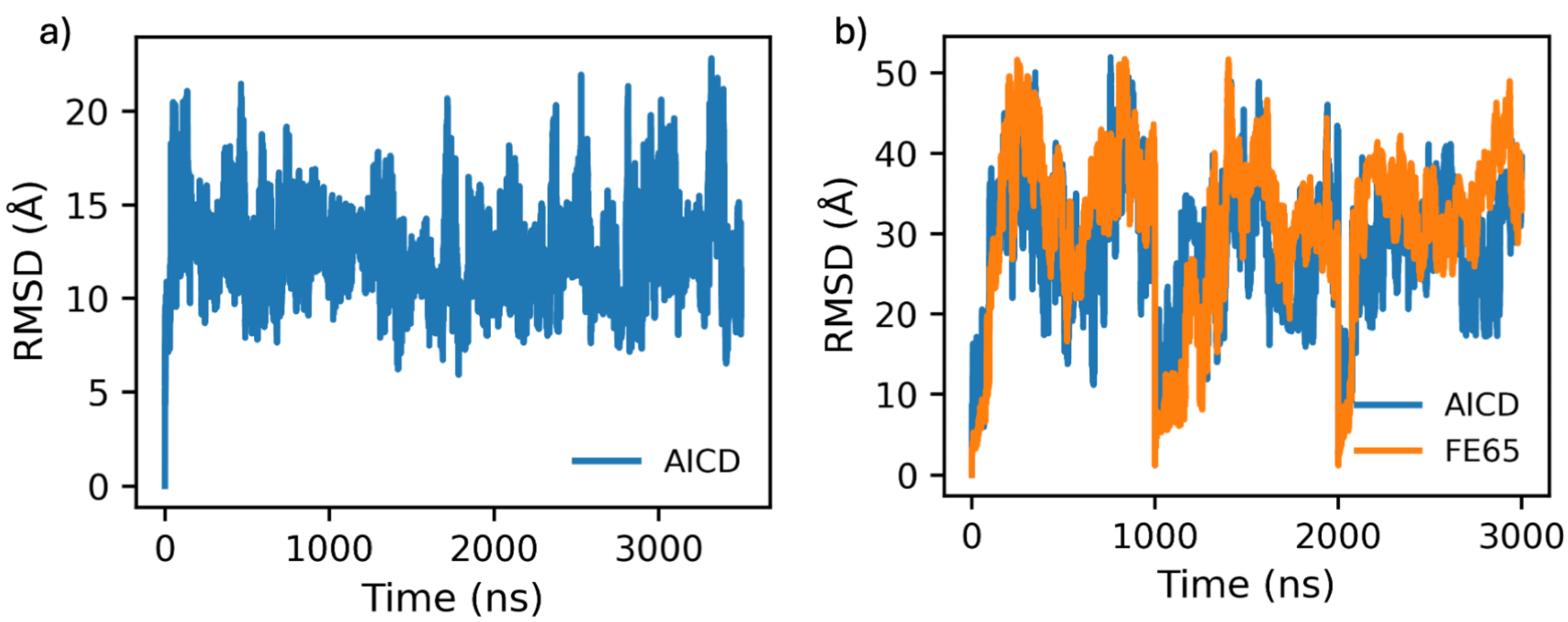

Figure S1: Backbone RMSD profiles from the MD simulations. (a) RMSD of isolated AICD as a function of simulation time. (b) RMSD of AICD and FE65 in the FE65–AICD complex, calculated relative to the corresponding starting coordinates. The traces indicate substantial conformational heterogeneity in both the free and bound states, with larger excursions observed for the components of the complex. RMSD is given in Å.

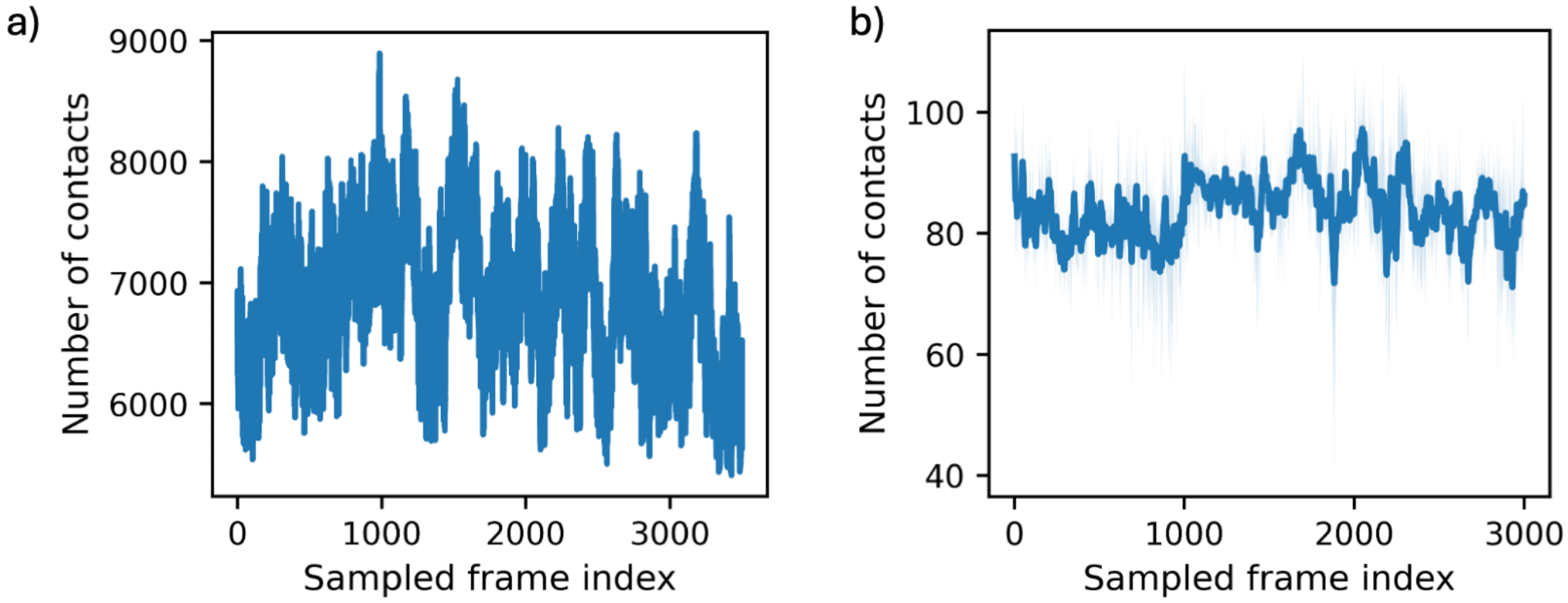

Figure S2: Time evolution of contact formation during the molecular dynamics trajectories. (a) Total number of intramolecular contacts as a function of sampled frame index, showing substantial fluctuations over the trajectory and indicating continuous rearrangement of the conformational ensemble. (b) Time evolution of the selected residue-residue contact population, shown with the raw contact signal in light blue and the smoothed trend in dark blue. The relatively persistent contact level in panel b indicates that this contact network remains maintained throughout the sampled trajectory, despite short-timescale fluctuations.

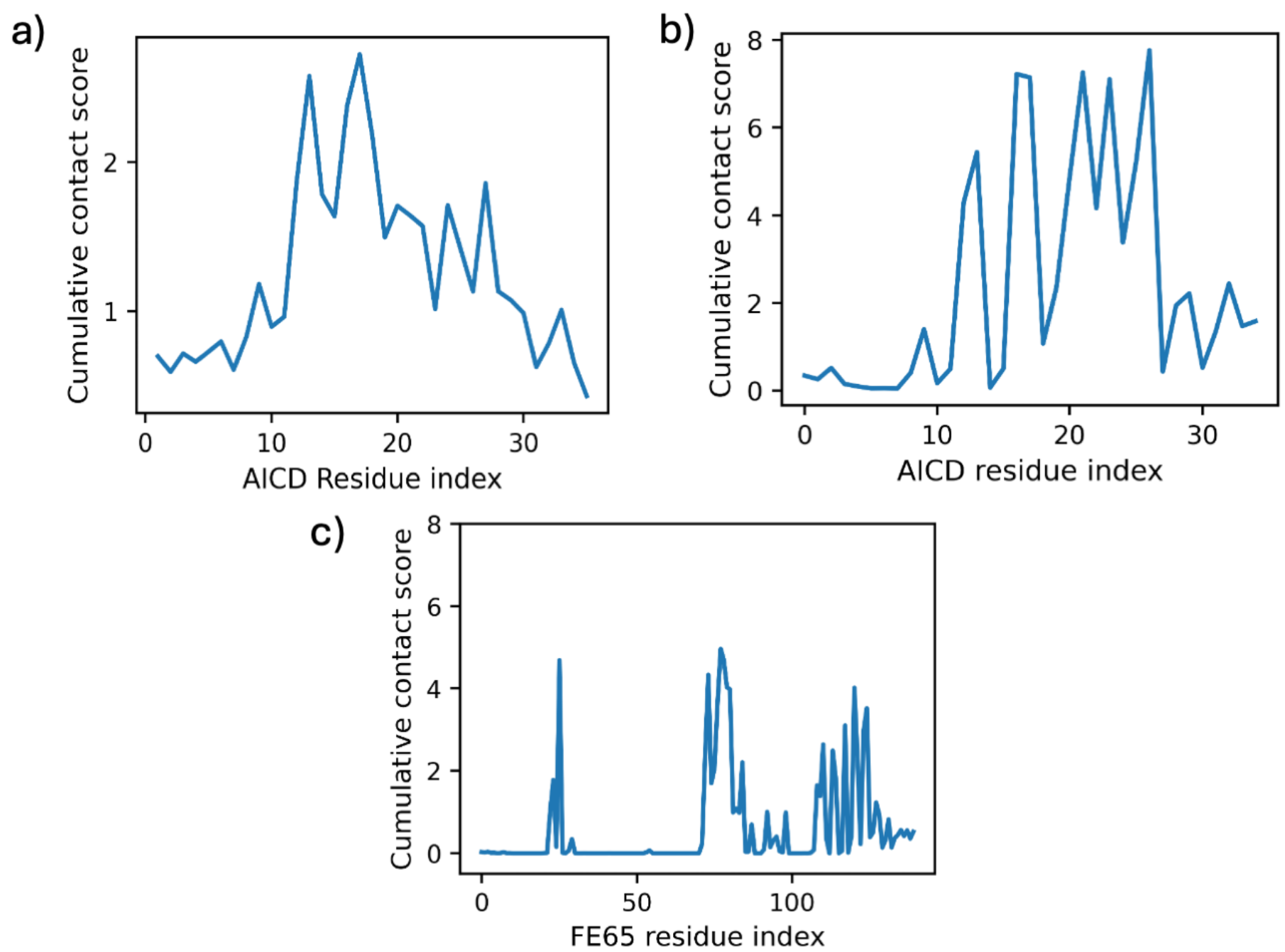

Figure S3: Residue-wise cumulative contact scores for isolated AICD and the FE65–AICD complex. (a) Cumulative intramolecular contact score for AICD in the unbound state. (b) Cumulative intermolecular contact score for AICD in the bound state. (c) Cumulative intermolecular contact score for FE65 in the bound state. The profiles identify the regions that contribute most strongly to internal organization in free AICD and to hotspot-mediated recognition in the FE65-bound complex.

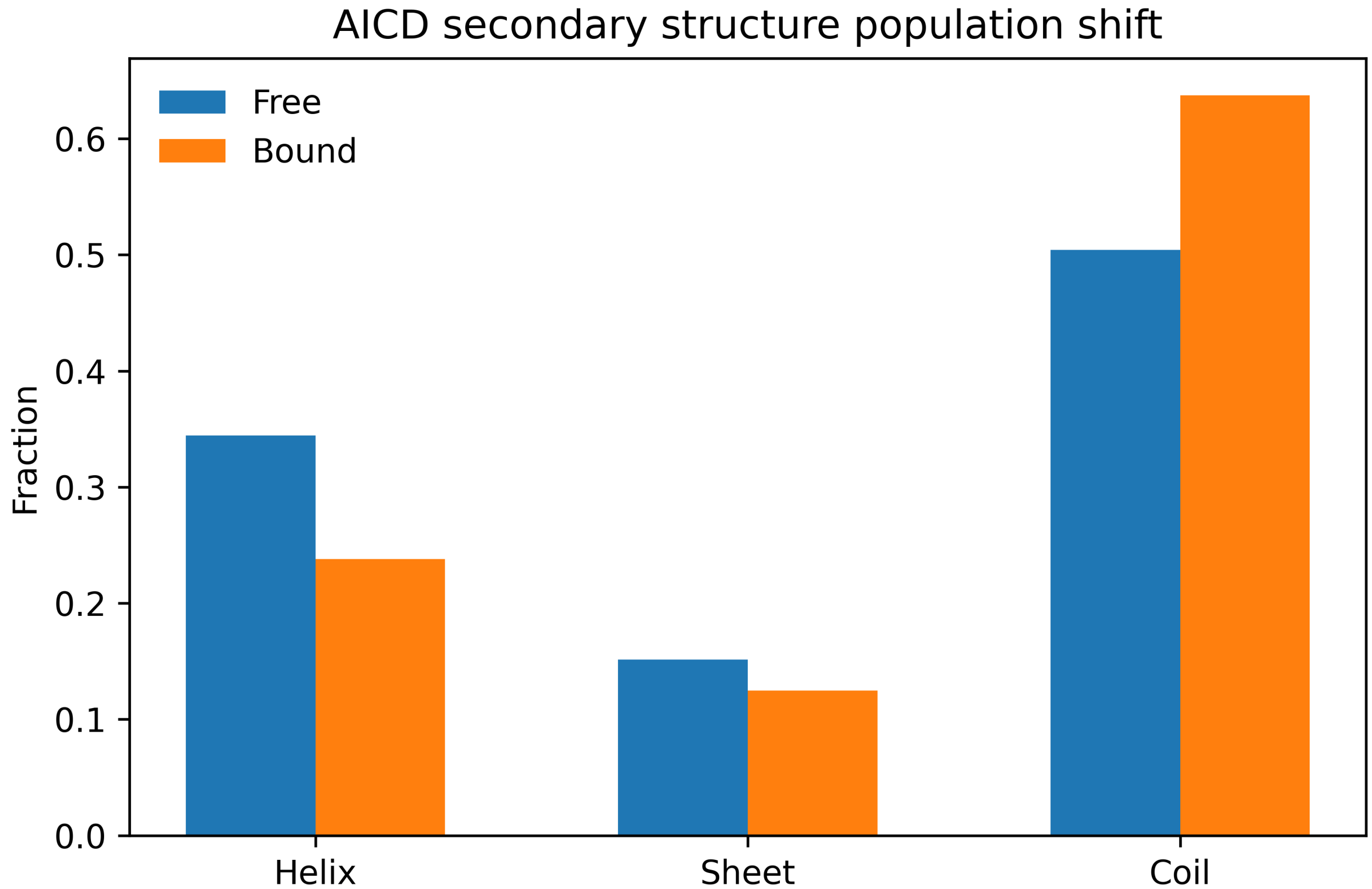

Figure S4: Secondary-structure population shift of AICD upon FE65 binding. Fractions of helix, sheet, and coil conformations are compared for free AICD and FE65-bound AICD. Free AICD displays higher helix and sheet populations, whereas the bound state shows an increased coil fraction. The decrease in ordered secondary-structure content upon binding indicates that FE65 association redistributes the AICD conformational ensemble rather than stabilizing a uniformly folded peptide structure.

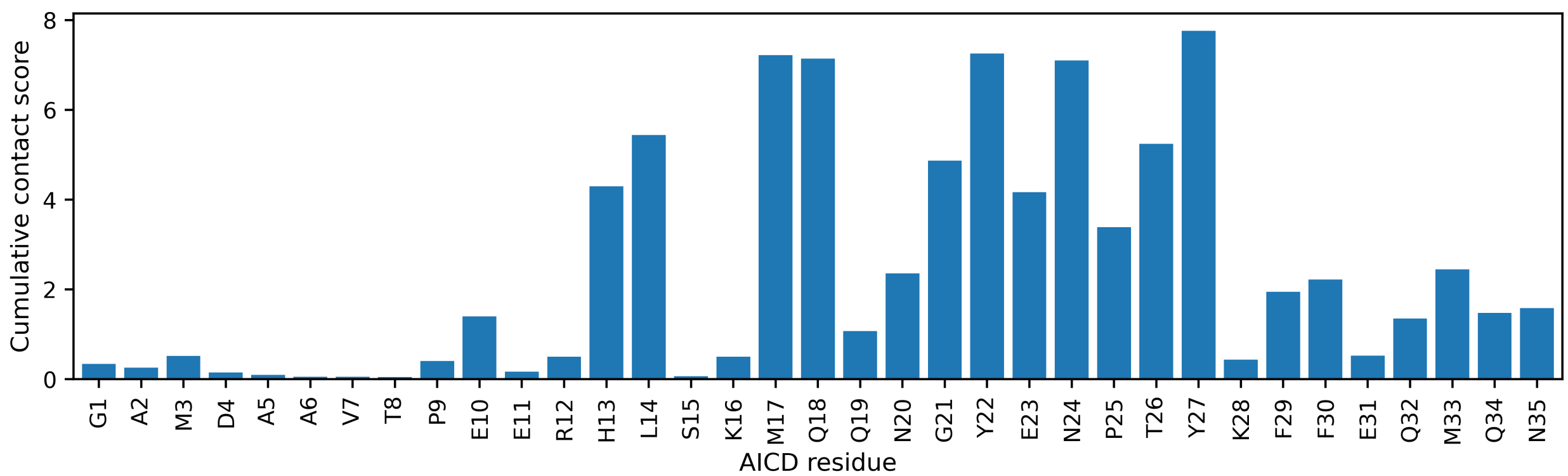

Figure S5: Residue-resolved contact hotspot profile of AICD. The cumulative contact score is plotted for each AICD residue, with larger values indicating residues that participate more frequently or persistently in contact formation during the trajectory. The strongest contributions are concentrated in the central-to-C-terminal region, including M17, Q18, Y22, N24, T26, and Y27, highlighting the importance of residues within and near the YENPTY motif in stabilizing the contact network.

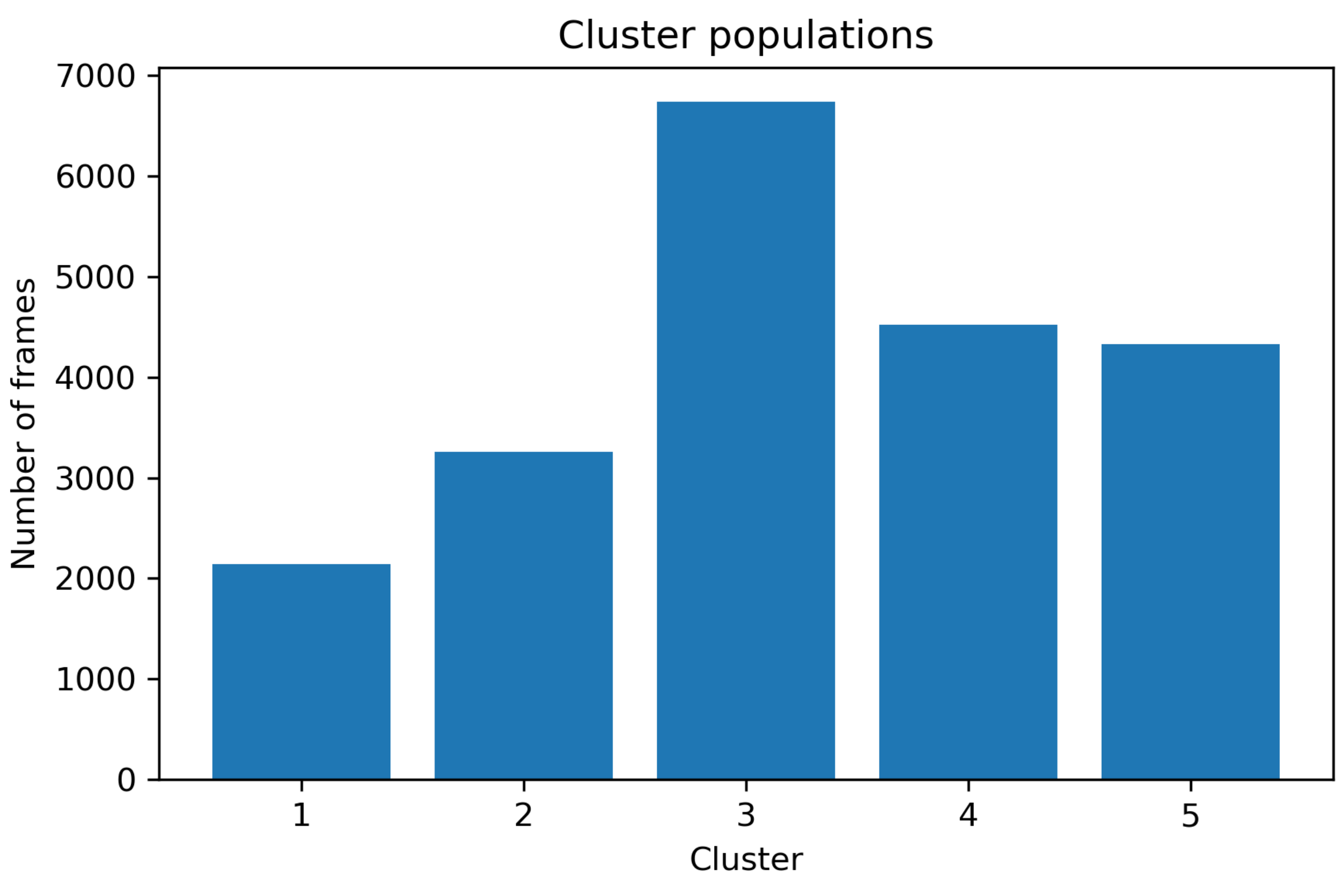

Figure S6: Population distribution of the conformational clusters obtained from the trajectory analysis. The number of frames assigned to each cluster is shown for clusters 1–5. Cluster 3 represents the most populated state, whereas cluster 1 is the least populated, indicating that the sampled ensemble is distributed among several substates with unequal statistical weights.
